# Auricular acupuncture for substance use: a randomized controlled trial of effects on anxiety, sleep, drug use and use of addiction treatment services

**DOI:** 10.1186/s13011-016-0068-z

**Published:** 2016-07-25

**Authors:** Rickard Ahlberg, Kurt Skårberg, Ole Brus, Lars Kjellin

**Affiliations:** Faculty of Medicine and Health, University Health Care Research Center, Örebro University, P.O. Box 1613, SE-701 16 Örebro, Sweden; Addiction Center, Faculty of Medicine and Health, Örebro University, P.O. Box 1613, SE-701 16 Örebro, Sweden; Clinical Epidemiology and Biostatistics, Faculty of Medicine and Health, Örebro University, P.O. Box 1613, SE-701 16 Örebro, Sweden

**Keywords:** Auricular acupuncture, Psychiatric comorbidity, Randomized controlled trial, Relaxation, Substance abuse treatment

## Abstract

**Background:**

A common alternative treatment for substance abuse is auricular acupuncture. The aim of the study was to evaluate the short and long-term effect of auricular acupuncture on anxiety, sleep, drug use and addiction treatment utilization in adults with substance abuse.

**Method:**

Of the patients included, 280 adults with substance abuse and psychiatric comorbidity, 80 were randomly assigned to auricular acupuncture according to the NADA protocol, 80 to auricular acupuncture according to a local protocol (LP), and 120 to relaxation (controls). The primary outcomes anxiety (Beck Anxiety Inventory; BAI) and insomnia (Insomnia Severity Index; ISI) were measured at baseline and at follow-ups 5 weeks and 3 months after the baseline assessment. Secondary outcomes were drug use and addiction service utilization. Complete datasets regarding BAI/ISI were obtained from 37/34 subjects in the NADA group, 28/28 in the LP group and 36/35 controls. Data were analyzed using Chi-square, Analysis of Variance, Kruskal Wallis, Repeated Measures Analysis of Variance, Eta square (η^2^), and Wilcoxon Signed Ranks tests.

**Results:**

Participants in NADA, LP and control group improved significantly on the ISI and BAI. There was no significant difference in change over time between the three groups in any of the primary (effect size: BAI, η^2^ = 0.03, ISI, η^2^ = 0.05) or secondary outcomes. Neither of the two acupuncture treatments resulted in differences in sleep, anxiety or drug use from the control group at 5 weeks or 3 months.

**Conclusion:**

No evidence was found that acupuncture as delivered in this study is more effective than relaxation for problems with anxiety, sleep or substance use or in reducing the need for further addiction treatment in patients with substance use problems and comorbid psychiatric disorders. The substantial attrition at follow-up is a main limitation of the study.

**Trial registration:**

Clinical Trials NCT02604706 (retrospectively registered).

## Background

The abuse of illicit psychoactive substances and alcohol is a major worldwide public health problem [1]. In Sweden, 6 % of the total population have a DSM-IV alcohol abuse and/or dependence diagnosis and 1.4 % of the population have a DSM-IV diagnosis of abuse of and/or dependence on illicit substances [2]. Many of those with an alcohol use disorder also have a drug use disorder and vice versa. Abuse of a single drug or alcohol alone is relatively rare among patients in substance abuse treatment [3]. Comorbidity between substance/alcohol abuse and other psychiatric disorders is common with 50 % having at least one more disorder. Anxiety, mood disorders and antisocial personality disorder are the most prevalent comorbid diagnoses [4]. Although there is some evidence that specific psychosocial interventions (e.g. Cognitive behavioral therapy; [5]) can reduce problems in patients with single substance use without psychiatric comorbidity, there is limited evidence to support any one intervention over another in the treatment of polysubstance abuse with psychiatric comorbidity [6, 7].

Alcoholism has been described at least since the ancient Greek and Roman times [8]. A wide variety of treatments for alcohol and drug use problems have been tried and are used in the standard care of patients with substance use problems, both pharmacological and psychological [5–7, 9, 10]. Several alternative treatments have also been tried, e.g. neurofeedback, art-based therapy, and eastern influenced treatments like yoga and meditation [11–13]. One of the more common alternative treatments for substance abuse is acupuncture, in particular auricular acupuncture. It has been reported that about seven percent of patients with substance abuse have tried acupuncture [14, 15]. Over 25 years of clinical experience has supported ear acupuncture and its proponents say it alleviates withdrawal, reduces craving, and helps retain patients in treatment [16]. A randomized study by Avant and colleagues found effects of auricular acupuncture on cocaine dependence [17]. However, several reviews have failed to find support for acupuncture as an effective treatment for substance abuse and dependence (e. g. cocaine abuse, alcohol dependence, and opioid addiction), although the poor methodological quality of the studies included has prevented any firm conclusions to be drawn [18–20]. These large reviews all suggests that more research on acupuncture with rigorous and large clinical trials are needed.

In the Swedish national clinical guidelines on substance abuse treatment from 2007 it was concluded that RCT-studies on acupuncture for substance use problems had not found any effect above placebo effects but that there could be effects on other problem areas [21]. White [22] suggested that the lack of effects of acupuncture in clinical trials could be due to the acupuncture technique used, and the choice of controls and outcome measures. White found that studies with sham controls were less likely to be positive than those with non-acupuncture controls, and positive results were more likely when using measures of craving or withdrawal than when measuring abstinence. In a systematic review and meta-analysis of the efficacy of acupuncture for psychological symptoms associated with opioid addiction, four studies from Western countries did not report any clinical gains in the treatment of these symptoms. Ten out of twelve studies from China did however report positive findings and found a significant difference between treatment groups and control groups for anxiety and depression associated with opioid addiction. The methodological quality of the studies included was considered poor [23]. The aim of the present study was to investigate the effectiveness of two versions of auricular acupuncture in a large randomized clinical trial. The main outcome measurements are anxiety and sleeping problems. Secondary outcomes are alcohol and drug use and utilization of addiction treatment services.

## Methods

### Setting and procedure

Data were collected between October, 2010, and June, 2014. Participants were recruited from a substance abuse clinic for people aged 16 years and above in Örebro, Sweden—the Addiction Center (AC)—with a catchment area of around 290,000 inhabitants. The clinic is linked to the University hospital in Örebro and serves about 880 unique inpatients and 1100 unique outpatients a year. In order to receive treatment at the AC patients have to have substance abuse and comorbid psychiatric problems, assessed and confirmed by psychologist and psychiatrist assessments and recurrent urine tests. Treatment at AC involves a mix of social, psychological, and medical therapies and interventions, e.g. pharmacological treatment in severe cases of depression and anxiety and for AD/HD and other mental disorders, Antabuse if required, manual based relapse prevention, Cognitive behavioral therapy, Psychodynamic therapy, Motivational Interviewing, and support from social workers.

A block randomization schedule with varying block sizes was created in the statistical software SPSS by a biostatistician, the third author (OB). The list was used to place participants who gave informed consent at random into one of three different groups: NADA (National Acupuncture Detoxification Association)-acupuncture, local protocol-acupuncture (LP), or control (relaxation). Based on clinical experience at the AC a larger dropout was expected among those who were randomly selected as controls than those allocated to acupuncture. The allocation ratio was NADA 2: LP 2: Control 3. Before start of patient inclusion, the second author (KS) prepared envelopes with code number and assigned intervention, sealed the envelopes and placed them in ascending order in a box.

Patients were invited to participate in the study by posters and orally during regular treatment sessions by receptionists and therapists at all AC units. Those who expressed interest were given more detailed information by the acupuncturists in the study, and were told that participation was voluntary, that the study was a randomized trial, and that the participants would be randomly selected for the usual treatment together with acupuncture or to be in a control group that would receive the usual treatment and relaxation. Those accepting participation signed a written informed consent form. The acupuncturist then contacted an assistant who drew the envelope in turn, opened it and revealed the assigned intervention. The assistant worked independently and had no other role in the study.

All groups were given self-report questionnaires immediately before the start of the treatment period (T1). Follow-up post-treatment data collection took place at 5 weeks (T2) and 3 months (T3) after initiation of the treatment. Patients randomly selected as controls were offered acupuncture after completing T3. The project was approved by the Regional Ethics Review Board in Uppsala, Sweden (Registration number 2010/239).

### Interventions

Participants who gave informed consent were randomly selected for one of three different treatments: NADA-acupuncture [24], local protocol-acupuncture (LP), or control (relaxation). NADA-acupuncture was delivered in three phases: (1) one treatment each workday during the first week; (2) three treatments each week during the following 2 weeks; (3) two treatments each week during another 2 weeks. The LP-acupuncture was delivered in two phases: (1) three treatments each week during the 2 first weeks; (2) two treatments each week for the following 2 weeks. This choice of treatment was based on about 15 years of clinical use of auricular acupuncture, from which both patients and acupuncturists had reported positive experiences. Relaxation consisted of listening to soft music in a quiet room with dampened light and was delivered to match the amount and phases of the LP-acupuncture. Within each group, there was no variation in treatment. The two acupuncture interventions thus comprised different number of sessions (15 in NADA and 10 in LP), all carried out individually in a separate room, but equal treatment: each session consisted of approximately 40 min retention time with acupuncture at five ear points called Sympathetic, Shen Men, Kidney, Liver and Lung, which are believed to be the best points for substance abuse patients [25]. Acupuncture was administered to both ears using stainless steel needles (0,25x13mm). The depth of insertion was 2–3 mm and manual needle stimulation was used. All three interventions were given as a supplement to treatment as usual (see ‘Setting and procedure’ above). Twelve male and female acupuncturists, all having gone through the same national training and thereby certified in NADA-acupuncture, administered NADA-acupuncture, the LP-acupuncture, and the relaxation. Their experience of practicing auricular acupuncture varied from 6 months to 20 years.

### Measurement

Anxiety was measured at treatment start and follow-up using the Beck Anxiety Inventory (BAI) [26], which has shown good reliability [27] and validity [28]. Sleep problems were measured at the same time points using the Insomnia Severity Index (ISI) which has shown god reliability and validity [29]. Alcohol use before treatment start was measured by the Alcohol Use Disorders Identification Test (AUDIT) [30], and drug use before treatment start by Drug Use Disorders Identification Test (DUDIT) [31]. AUDIT and DUDIT have good psychometric properties [30, 31]. The Drug Use Disorders Identification Test-Extended (DUDIT-E) [32], with added items to measure use of alcohol and anabolic androgenic steroids, was used in follow-up assessments.

Diagnoses (the main diagnosis recorded closest in time to start of intervention) according to ICD-10 as well as data on outpatient visits to a doctor and inpatient treatment episodes at the AC 6 months before and 6 months after treatment initiation were gathered from the clinical files. For subjects who were inpatients when treatment started, the episode in question were counted as an admission before start of treatment while the inpatient days of this episode were split and entered as either prior to or after the date treatment started.

### Power calculation

A power calculation was performed assuming a clinically relevant difference between the groups of six BAI units [33] and a standard deviation of 10.49. Further, a significance level of 95 % and a power of 80 % were used. From the relaxation group a dropout of 60 % was assumed and from the two other treatment arms 40 %. The higher dropout rate from relaxation group was due to an assumption that patients included wanted acupuncture, and that those who were randomized to the relaxation group would be more likely to drop out. This resulted in a total of 315 individuals needed to be included.

### Participants

**Fig. 1:**
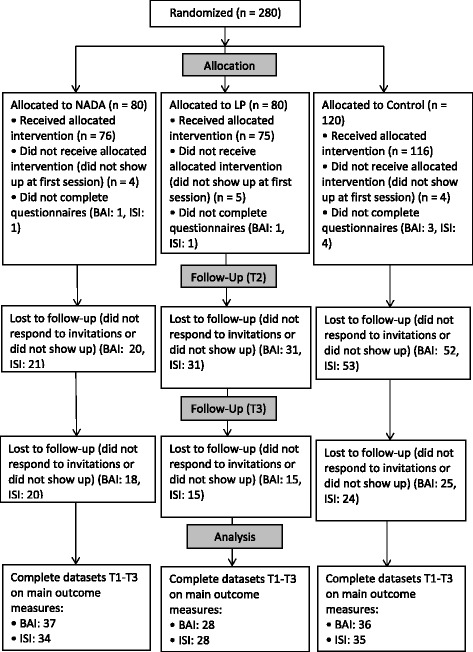
Flow of participants

Participants in the study were in treatment for substance abuse and psychiatric comorbidity at the AC. Both inpatients and outpatients were recruited. Inclusion criteria were: (1) 18–65 years of age, and (2) ongoing patient status at the AC. Exclusion criteria were (1) nickel-allergy, (2) ear infection, and (3) heart disease. On the basis of these criteria 280 patients were recruited to participate in the study and allocated at random to one of the three interventions. A few patients dropped out before starting the treatment, and 267 received their allocated intervention. The flow of participants in the study is presented in Fig. 1. Data on relapse in alcohol use or not were obtained from 163 participants at T2 and 120 at T3, and answers about the use of other drugs from 153 at T2 and 115 at T3. In many cases participants gave no reasons for not showing up to a treatment session or for terminating their participation in the study. In cases when reasons were recorded, the most frequent were illness, followed by work, lack of time, delay, family reasons, and relapse into substance use.

### Statistics

Data were analyzed using the IBM SPSS Statistics for Windows statistical package, version 22.0. Differences in categorical variables between patients allocated to NADA, LP and control respectively were analyzed using Chi-square tests. Age, number of sessions, and baseline performance of the three groups on BAI, ISI, AUDIT and DUDIT were analyzed with Analysis of Variance (ANOVA). Cases with missing values for up to three BAI items, one ISI item, two AUDIT items and two DUDIT items were included in the analyses. In these cases, missing values were imputed as values equal to the individual case mean of the completed items. Due to skewed distributions, service use data for the three treatment groups were analyzed using the Kruskal Wallis test. Treatment effects for anxiety and sleeping problems were analyzed with Repeated Measures Analysis of Variance with time as a within-subjects factor and group as a between-subjects factor. Effect sizes were measured using eta square (η^2^). In order to look at the in- and outpatients separately a sub-analyses of repeated measurements ANOVA were preformed stratified on type of care. Treatment effects for alcohol and drug use were analyzed using a Chi-square test or Fisher’s exact test when appropriate. For comparisons of service use before and after start of treatment respectively, the Wilcoxon Signed Ranks test was used. *P*-values <0.05 were considered statistically significant.

## Results

Fourtyfour per cent of the participants were women and their mean age was 44.5 years. The main diagnosis for more than 50 % of the participants was mental and behavioral disorders due to use of alcohol, and 38 % were inpatients at the start of treatment. There were no differences between treatment groups at T1 with regard to gender, age, service use at the AC 6 months before start of treatment, inpatient status, main diagnosis and BAI, ISI, AUDIT and DUDIT scores. The mean number of attended sessions was lower than intended for each intervention, but participants allocated to the longest treatment, NADA, received, as intended, more treatment sessions on average than those given acupuncture according to LP or relaxation (Table 1).

**Table 1:** Patient characteristics and number of acupuncture or relaxation sessions attended

	NADA	Local Protocol	Control	Total	*p*
	*n* = 80	*n* = 80	*n* = 120	*n* = 280	
Female gender, %	37.5	41.3	50.0	43.9	0.185
Age, M(SD)	44.1(14.0)	44.3(14.2)	44.8(13.0)	44.5(13.6)	0.924
6 months before start of treatment, M(SD):					
Visits to the doctor	1.5(1.7)	2.1(2.0)	1.8(2.0)	1.8(1.9)	0.062
Inpatient admissions	0.7(0.8)	0.9(1.1)	0.7(0.7)	0.7(0.9)	0.406
Inpatient days	2.6(4.6)	3.8(7.8)	3.3(7.3)	3.2(6.8)	0.705
Inpatient at treatment start, %	35.0	45.0	35.8	38.2	0.333
Main diagnosis, %	*n* = 73	*n* = 80	*n* = 108	*n* = 261	
Mental and behavioral disorders due to: use of alcohol, F10.1-10.3	52.1	48.8	60.2	54.4	–
use of opioids, cannabinoids, sedatives or hypnotics, F11.1–13.2	23.3	17.5	17.6	19.2	0.649
multiple drug use, F19.1–19.2	15.1	18.8	10.2	14.2	–
Other psychiatric diagnosis, F29.2–90.0	8.2	12.5	11.1	10.7	–
General psychiatric examination, Z00.4	1.4	2.5	0.9	1.5	–
	*n* = 75	*n* = 74	*n* = 113	*n* = 262	
BAI sum score at T1, M(SD)	21.7(12.5)	19.9(10.8)	21.6(11.9)	21.1(11.8)	0.557
	*n* = 75	*n* = 74	*n* = 112	*n* = 261	
ISI sum score at T1, M(SD)	15.4(7.2)	13.9(7.3)	14.4(6.9)	14.6(7.1)	0.390
	*n* = 72	*n* = 72	*n* = 109	*n* = 253	
AUDIT at T1, M(SD)	17.3(12.3)	18.6(11.9)	19.0(12.0)	18.4(12.0)	0.637
	*n* = 74	*n* = 71	*n* = 112	*n* = 257	
DUDIT at T1, M(SD)	10.5(14.5)	10.1(13.8)	9.0(13.2)	9.7(13.7)	0.730
	*n* = 68	*n* = 67	*n* = 100	*n* = 235	
Number of sessions, M(SD)	11.9(4.7)	7.1(3.4)	6.9(3.9)	8.4(4.6)	<0.001

When comparing those who completed questionnaires at T3 (*n* = 120) with those who dropped out between randomization and T3 (*n* = 160), there were no differences in gender, diagnosis, and BAI, ISI, AUDIT and DUDIT scores. Participants reassessed at T3 were older (mean [sd] 47.0[13.4] vs. 42.5[13.5], t = 2.77, df = 278, *p* = 0.006), were more frequently inpatients (55.8 % vs. 25.0 %, Chi-square = 27.61, df = 1, *p* < 0.001) and completed more sessions (mean [sd] 10.0[3.7] vs. 6.9[4.8], t = 5.57, df = 233, *p* < 0.001) than participants who dropped out before T3.

**Fig. 2:**
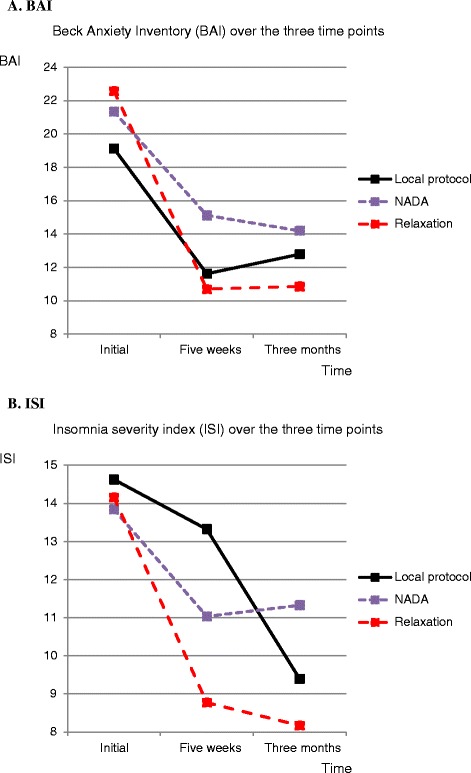
Mean scores at T1, T2 and T3 for Beck Anxiety Inventory (BAI) and Insomnia Severity Index (ISI)

Outcome data from the baseline and post-treatment BAI and ISI are presented in Fig. 2 and Table 2. The interaction effects of group and time in the repeated measurement ANOVA were not significant, neither in BAI (F[1.45, 3.13], *p* = 0.229, η^2^ = 0.03, NADA decreased 7.2 points between T1 and T3, LP decreased 6.3 points between T1 and T3 and Control decreased 11.7 points between T1 and T3) or ISI(F[2.27, 4], *p* = 0.065, η^2^ = 0.05, NADA decreased 2.5 units, LP 5.2 units and control 6.0 units). There were significant time effects for both BAI (F[32.66, 1.56], *p* < 0.001), η^2^ = 0.25 and ISI (F[18.06, 2], *p* < 0.001), η^2^ = 0.16. There were no significant group differences (BAI: F[0.57, 2], *p* = 0.569, η^2^ = 0.01, ISI: F[0.95, 2], *p* = 0.392), η^2^ = 0.02.

**Table 2:** Mean (standard deviation) raw scores at T1, T2 and T3 for Beck Anxiety Inventory (BAI) and Insomnia Severity Index (ISI)

	NADA	Local protocol	Control
BAI^a^	*n* = 37	*n* = 28	*n* = 36
T1	21.4 (14.2)	19.1 (13.2)	22.6 (14.0)
T2	15.1 (12.9)	11.6 (9.0)	10.7 (10.5)
T3	14.2 (12.7)	12.8 (13.6)	10.9 (10.6)
ISI ^b^	*n* = 34	*n* = 28	*n* = 35
T1	13.8 (7.8)	14.6 (7.6)	14.2 (7.2)
T2	11.0 (7.3)	13.3 (7.5)	8.8 (8.6)
T3	11.3 (8.9)	9.4 (8.2)	8.2 (7.6)

^a^In the repeated measurements ANOVA for BAI the Interaction effect was: (F[1.45, 3.13], *p* = 0.229), Group effect: BAI (F[0.57, 2], *p* = 0.569) and Time effect: BAI (F[32.66, 1.56], *p* < 0.001)

^b^In the repeated measurements ANOVA for ISI the Interaction effect was: (F[2.27, 3.94], *p* = 0.065), Group effect: F[0.95, 2], *p* = 0.392) and Time effect: BAI (F[18.06, 1.97], *p* < 0.001)

When looking at a sub-analysis for inpatients and outpatients separately for BAI neither interaction effect was significant (for inpatients: F[1.92, 2.86], *p* = 0.137, η^2^ = 0.07, outpatients: F[1.06, 3.76], *p* = 0.383, η^2^ = 0.05) or group effect (inpatients: F[0.46, 2], *p* = 0.636, η^2^ = 0.02, outpatients: F[2.55, 2], *p* = 0.091, η^2^ = 0.11) but a significant time effect (inpatients: F[26.59,1.43], *p* < 0.001, η^2^ = 0.33, outpatients: F[5.88, 1.88], *p* = 0.005, η^2^ = 0.13). For ISI there was a significant interaction effect for inpatients, but not outpatients (inpatients: F[3.27, 3.94], *p* = 0.015, η^2^ = 0.11, outpatients: F[1.16, 3.99], *p* = 0.336, η^2^ = 0.06). There was a time effect for both types of care (inpatients: F[16.47, 1.97], *p* < 0.001, η^2^ = 0.24, outpatients: F[3.61, 2.00], *p* = 0.032, η^2^ = 0.09), but no group effect (inpatients: F[1.98, 2], *p* = 0.148, η^2^ = 0.07, outpatients: F[1.22, 2], *p* = 0.308, η^2^ = 0.06).

Around nine to twelve per cent of the participants reported that they had relapsed in alcohol use or used at least one other drug at T2 and T3. There were no statistically significant differences in this respect between those who had received NADA-acupuncture, acupuncture according to the local protocol, or relaxation (Table 3).

**Table 3:** Relapse in alcohol use and use of drugs at T2 and T3

	NADA	Local protocol	Control	Total	*p*
Relapse in alcohol use, %	*n* = 55	*n* = 43	*n* = 65	*n* = 163	
T2	12.7	11.6	12.3	12.3	0.986
	*n* = 41	*n* = 34	*n* = 45	*n* = 120	
T3	7.3	14.7	11.1	10.8	0.590
Use of at least one drug^a,^ %	*n* = 50	*n* = 41	*n* = 62	*n* = 153	
T2	8.0	9.8	11.3	9.8	0.844
	*n* = 40	*n* = 32	*n* = 43	*n* = 115	
T3	12.5	9.4	4.7	8.7	0.442

^a^Cannabis, amphetamine, cocaine, opiates, hallucinogenic or other drugs (alcohol excluded)

Comparison of service use at the AC 6 months before and 6 months after start of treatment, showed that inpatient admissions decreased for all groups while inpatient days increased for both acupuncture groups. There were no changes in the number of visits to the doctor for any of the groups (Table 4).

**Table 4:** Visits to the doctor and inpatient admissions and days at the Addiction Center 6 months before and 6 months after start of treatment

	NADA	Local protocol	Control	Total
	*n* = 80	*n* = 80	*n* = 120	*n* = 280
Visits to the doctor				
6 months before	1.5(1.7)	2.1(2.0)	1.8(2.0)	1.8(1.9)
6 months after	1.5(1.8)	2.0(2.9)	1.9(3.4)	1.8(2.8)
*p*	0.651	0.085	0.830	0.365
Inpatient admissions				
6 months before	0.7(0.8)	0.9(1.1)	0.7(0.7)	0.7(0.9)
6 months after	0.4(0.7)	0.5(1.2)	0.4(0.7)	0.4(0.9)
*p*	0.002	0.005	<0.001	<0.001
Inpatient days				
6 months before	2.6(4.6)	3.8(7.8)	3.3(7.3)	3.2(6.8)
6 months after	5.9(10.5)	7.3(11.4)	4.3(8.4)	5.6(10.0)
*p*	0.006	0.010	0.221	<0.001

Mean(standard deviation)

## Discussion

The aim of the current study was to investigate the short and long-term effects of two versions of auricular acupuncture, NADA-acupuncture and a local acupuncture protocol adapted from the NADA protocol, on anxiety symptoms, sleeping problems, substance use and addiction service use among psychiatric patients with substance use problems. The two treatment conditions were compared with relaxation. The results indicate that symptoms of anxiety and sleeping problems showed both short and long term improvement. There were no significant interaction effects for either BAI or ISI, suggesting that improvements in anxiety symptoms and sleeping problems were comparable across the three groups and effect sizes were small. Patients in all the three groups started on average with moderate to severe levels of anxiety at baseline as rated by the BAI, and all groups lowered the mean score from T1 to T3 to the mild to moderate range [34]. Patients in all three groups started on average at the border of sub-clinical insomnia/moderate insomnia as rated by the ISI, and lowered to the lowest level of sub-threshold insomnia just above the score for absence of insomnia [35]. Our findings are consistent with research showing that non-specific treatment factors and the simple provision of support have positive effects on psychiatric symptoms [36, 37]. It is also plausible that some of the effects in all three groups are effects of regression to the mean [38]. Another possibility is that both acupuncture and relaxation have effects on anxiety and sleeping problems. In a pilot study of veterans recovering from substance use disorders by Chang and colleagues, in which study participants were randomly assigned to acupuncture, relaxation response training or TAU, it was found that both the acupuncture and the relaxation groups had greater improvements in anxiety levels than the TAU group [39].

Those assigned to relaxation in our study did however not get an actual relaxation training intervention as the patients in the study by Chang and colleagues mentioned above. The relaxation intervention in our study consisted of listening to music in a quiet room with a dampened light. We are not aware of any randomized studies that have found long-term effects of music listening on anxiety and sleeping problems, and we suggest therefore that the most plausible interpretation is that the effects found in our study are non-specific effects. There are however studies that have found effects of acupuncture on other outcomes. Stuyt & Meeker [40] found in a naturalistic study on auricular acupuncture that patients receiving needles reported significant improvement in anger, concentration and pain management. Carter et al. [41] found that NADA-acupuncture had significant effects on body aches, cravings and energy. These two studies were non-randomized, limiting the evidence of actual effects. Chang et al. [39] found significant effects of acupuncture but not of relaxation on cravings in their randomized study. The acupuncture group did however receive twice as many intervention sessions as the relaxation group making the interpretation of effects difficult.

With regard to substance use, we found no differences between groups at follow-up. This finding is in agreement with earlier reviews [18–20, 42] who failed to find evidence of effects on substance abuse following acupuncture. Only about ten per cent of the patients in our study reported use of alcohol and/or other drugs at T3. Those who relapsed in drug use are probably over-represented among the drop-outs. Another explanation for the low relapse figures may be that being drug free is a requirement for receiving treatment at the AC.

All groups had on average fewer inpatient admissions during 6 months after start of treatment compared to before, while the number of inpatient days increased significantly for both acupuncture groups. The increase in inpatient days for all three groups in aggregate may be due to the fact that a relatively large proportion of the research subjects were inpatients when treatment started and that many of the interventions may have started at the beginning of the treatment episode. Our sub-analyses showed that for BAI there were no clear differences between in- and outpatients in how they change from inclusion to follow-up and the corresponding effect sizes were small. For ISI there was such a difference for the inpatients, but not for the outpatients. Therefore the change in sleep problems over time among inpatients seems to differ for the different treatment groups.

The current study has two major strengths. First, the treatments were implemented with a relatively unselected sample of inpatients and outpatients at a regular substance abuse clinic, which means that the study participants had high degree of comorbidity and relatively low adherence to the treatment provided. In other words, the study probably has high external validity. Second, the study design included three different conditions, one being relaxation/not acupuncture, allowing us to control partly for non-specific therapy factors (therapeutic alliance, contact time, and treatment credibility) in the acupuncture conditions. One of the strengths of the study is also a limitation: patients with substance abuse and high degree of comorbidity are renowned for relapses and low adherence to treatment. Fifty-seven per cent of the patients had dropped out by the time of the 3-month follow-up. Although large dropout rates are common in trials of interventions for patients with substance abuse [43], their extent limits interpretation of the results. In our study, those who dropped out were younger, more often outpatients and, as expected, completed fewer sessions than those remaining at T3. That acupuncture was given individually and not in a group setting, that few participants actually received the full amount of acupuncture according to the treatment protocols, and that we do not have data on patients assessed for eligibility and excluded before randomization are other limitations, but a consequence of the fact that the interventions were tested in a naturalistic setting.

The imputation method used (mean imputation) assumes that the questions a participant does not answer would have been answered like those that were answered. Other imputation methods could have been used, but most imputation methods have the same problem: they assume that the missing data approximately follows a pattern that in some way follow the rest of the data.

A further limitation is that we, since patient inclusion went slower than expected and we did not have funding to continue, had to finish data collection before we had reached the number of patients needed according to our power calculation. Although a larger sample may have detected statistically significant effects of acupuncture relative to relaxation training on some of the measures, the probability of such a finding can be questioned since the actual changes in BAI and ISI mean scores between T1 and T3 were greater in the control group than among those receiving acupuncture. We did not correct for multiple testing, but given our results, doing so would not have changed our conclusions.

## Conclusions

Bearing the limitations of the study discussed above in mind, we found in conclusion no evidence for acupuncture as delivered in this study being more effective than relaxation for problems with anxiety, sleep or substance use or in reducing the need for further addiction treatment in patients with substance use problems and comorbid psychiatric disorders. The failure to find effects of acupuncture over and above the simple provision of music listening in a quiet environment (the relaxation control condition) in this randomized controlled trial raises questions about the clinical use of acupuncture in patients with substance use.

## Abbreviations

AC, Addiction Center, Örebro, Sweden; AUDIT, Alcohol Use Disorders Identification Test; BAI, Beck Anxiety Inventory; DUDIT, Drug Use Disorders Identification Test; DUDIT-E, Drug Use Disorders Identification Test-Extended; ISI, Insomnia Severity Index; LP, Local Protocol; NADA, National Acupuncture Detoxification Association.

## References

[1] WHO (2010). Atlas on substance use: resources for the prevention and treatment of substance use disorders.

[2] Ramstedt M, Sundin E, Landberg J, Raninen J. ANDT-bruket och dess negativa konsekvenser i den svenska befolkningen 2013—en studie med fokus på missbruk och beroende samt problem för andra än brukaren relaterat till alkohol, narkotika, doping och tobak. [In Swedish]. Stockholm: STAD-rapport 55; 2014.

[3] Hartzler B, Donovan DM, Huang Z (2011). Rates and influences of alcohol use disorder comorbidity among primary stimulant misusing treatment-seekers: meta-analytic findings across eight NIDA CTN trials. Am J Drug Alcohol Abuse.

[4] Van Emmerik-van Oortmersen K, van de Glind G, Koeter MWJ, Allsop S, Auriacombe M, Barta C (2013). Psychiatric comorbidity in treatment seeking substance use disorder patients with and without ADHD: results of the IASP study. Addiction.

[5] Mchugh RK, Hearon BA, Otto MW (2010). Cognitive-behavioral therapy for substance use disorders. Psychiatr Clin North Am.

[6] Hunt GE, Siegfried N, Morley K, Sithartan T, Cleary M (2013). Psychosocial interventions for people with both severe mental illness and substance misuse. Cochrane Database Syst Rev.

[7] Klimas J, Field CA, Cullen W, O’Gorman CS, Glynn LG, Keenan E (2013). Psychosocial interventions to reduce alcohol consumption in concurrent problem alcohol and illicit drug users. Cochrane Database Syst Rev.

[8] Leibowitz JO (1967). Studies in the history of alcoholism—II. Acute alcoholism in ancient Greek and roman medicine. Br J Addict Alcohol Other Drugs.

[9] Marshall K, Gowing L, Ali R, Le Foll B. Pharmacotherapies for cannabis dependence. Cochrane Database Syst Rev. 2014;(12):CD008940. 10.1002/14651858.CD008940.pub2 PMC4297244 25515775

[10] Rösner S, Hackl-Herrwerth A, Leucht S, Vecchi S, Srisurapanont M. Opioid antagonists for alcohol dependence. Cochrane Database Syst Rev. 2010;8(12):CD001867. doi:10.1002/14651858.CD001867.pub2. 10.1002/14651858.CD001867.pub3 PMC13590218 21154349

[11] Saxby E, Peniston EG (1995). Alpha-theta brainwave neurofeedback training: an effective treatment for male and female alcoholics with depressive symptoms. J Clin Psychol.

[12] Aletraris L, Paino M, Edmond MB, Roman PM, Bride BE (2014). The use of art and music therapy in substance abuse treatment programs. J Addict Nurs.

[13] Alexander CN, Robinson P, Maxwell R (1994). Treating and preventing alcohol, nicotine, and drug abuse through transcendental meditation: a review and statistical meta-analysis. Alcohol Treat Q.

[14] Quan H, Lai D, Johnson D, Verhoef M, Musto R (2008). Complementary and alternative medicine use among Chinese and white Canadians. Can Fam Physician.

[15] Manheimer E, Anderson BJ, Stein MD (2003). Use and assessment of complementary and alternative treatments by intravenous drug users. Am J Drug Alcohol Abuse.

[16] Otto KC (2003). Acupunture and substance abuse: a synopsis, with indications for further research. Am J Addict.

[17] Avants KS, Margolin A, Holford TR, Kosten TR (2000). A randomized controlled trial of auricular acupuncture for cocaine dependence. Arch Intern Med.

[18] Cho SH, Wang WW (2009). Acupuncture for alcohol dependence: a systematic review. Alcohol Clin Exp Res.

[19] D’Alberto A (2004). Auricular acupuncture in the treatment of cocaine/crack abuse: a review of the efficacy, the use of the national acupuncture detoxification association protocol, and the selection of sham points. J Altern Complement Med.

[20] Ter Riet G, Kleijnen J, Knipshild P (1990). A meta-analysis of studies into the effect of acupuncture on addiction. Br J Gen Pract.

[21] Socialstyrelsen (2007). Nationella riktlinjer för missbruks- och beroendevård vägledning för socialtjänstens och hälso- och sjukvårdens verksamhet för personer med missbruks- och beroendeproblem.

[22] White A (2013). Trials of acupuncture for drug dependence: a recommendation for hypotheses based on the literature. Acupunct Med.

[23] Boyuan Z, Yang C, Xueyoung S, Sheng L (2014). Efficacy for psychological symptoms associated with opiod addiction: a systematic review and meta-analysis. Evid Based Complement Alternat Med.

[24] Smith M (1979). Acupuncture and natural healing in drug detoxification. Am J Acupunct.

[25] Mclellan AT, Grossman DS, Blaine JD, Haverkos HW (1993). Acupuncture treatment for drug abuse: a technical review. J Subst Abuse Treat.

[26] Beck AT, Epstein N, Brown G, Steer RA (1998). An inventory for measuring clinical anxiety: psychometric properties. J Consult Clin Psychol.

[27] Beck AT, Brown GK, Steer RA, Kuyken W, Grisham J (2001). Psychometric properties of the beck self-esteem scales. Behav Res Ther.

[28] Kohn PM, Kantor L, Decicco TL, Beck AT (2008). The beck anxiety inventory-trait (BAIT): a measure of dispositional anxiety not contaminated by dispositional depression. J Pers Assess.

[29] Bastien CH, Valliéres A, Morin CM (2001). Validation of the insomnia severity index as an outcome measure for insomnia research. Sleep Med.

[30] Bergman H, Källmén H (2002). Alcohol use among Swedes and a psychometric evaluation of the alcohol use disorders identification test. Alcohol Alcohol.

[31] Berman AH, Bergman H, Palmstierna T, Schlyter F (2005). Evaluation of the drug use disorders identification test (DUDIT) in criminal justice and detoxification settings and in a Swedish population sample. Eur Addict Res.

[32] Berman AH, Palmstierna T, Källmén H, Bergman H (2007). The self-report drug use disorders identification test-extended (DUDIT-E): reliability, validity, and motivational index. J Subst Abuse Treat.

[33] Muntingh A, Feltz-Cornelis C, van Marwijk H, Spinhoven P, Assendelft W, de Waal M (2009). Collaborative stepped care for anxiety disorders in primary care: aims and design of a randomized controlled trial. BMC Health Serv Res.

[34] Julian LJ (2011). Measures of anxiety: state-trait anxiety inventory (STAI), beck anxiety inventory (BAI), and hospital anxiety and depression scale-anxiety (HADS-a). Arthritis Care Res.

[35] Morin CM, Belleville G, Bélanger L, Ivers H (2011). The insomnia severity index: psychometric indicators to detect insomnia cases and evaluate treatment response. Sleep.

[36] Horwath AO, Seymonds BD (1991). Relation between working alliance and outcome in psychotherapy: a meta-analysis. J Couns Psychol.

[37] Khan A, Faucett J, Lichtenberg P, Kirsch I, Brown WA (2012). A systematic review of comparative efficacy of treatments and controls for depression. PLoS One.

[38] Stigler SM (1997). Regression to the mean, historically considered. Stat Methods Med Res.

[39] Chang BH, Sommers E, Herz L (2010). Acupuncture and relaxation response for substance use disorder recovery. J Subst Use.

[40] Stuyt EB, Meeker JL. Benefits of auricular acupuncture in tobacco-free inpatient dual diagnosis treatment. J Dual Diagn. 2006;2:41–52.

[41] Carter KO, Olshan-Perlmutter M, Norton JJ, Smith MO (2011). NADA acupuncture perspective trial in patients with substance use disorders and seven common health symptoms. Med Acupunct.

[42] Bullock ML, Kiersuk TJ, Sherman RE, Lenz SK, Culliton PD, Boucher TA (2002). A large randomized placebo controlled study of auricular acupuncture for alcohol dependence. J Subst Abuse Treat.

[43] Heather N (2014). Interpreting null findings from trials of alcohol brief interventions. Front Psychiatry.

